# Investigation of the Energy Band at the Molybdenum Disulfide and ZrO_2_ Heterojunctions

**DOI:** 10.1186/s11671-018-2825-6

**Published:** 2018-12-17

**Authors:** Xinke Liu, Cong Hu, Kuilong Li, Wenjia Wang, Zhiwen Li, Jinping Ao, Jing Wu, Wei He, Wei Mao, Qiang Liu, Wenjie Yu, Ren-Jei Chung

**Affiliations:** 1 College of Materials Science and Engineering, Shenzhen Key Laboratory of Micro-scale Optical Information Technology, Guangdong Research Center for Interfacial Engineering of Functional Materials, Shenzhen University, 3688 Nanhai Ave, Shenzhen, 518060 People’s Republic of China; 2 School of Electronic and Information Engineering (Department of Physics), Qilu University of Technology (Shandong Academy of Sciences), Jinan, 250353 People’s Republic of China; 3 Institute of Materials research and Engineering (IMRE), 2 Fusionopolis Way, Innovis, #08-03, Singapore, 138634 Singapore; 4 College of Electronic Science and Technology, Shenzhen University, Shenzhen, 518060 People’s Republic of China; 5 The Institute of Engineering Innovation, School of Engineering, The University of Tokyo, Tokyo, 113-0032 Japan; 6 State Key Laboratory of Functional Materials for Informatics, Shanghai Institute of Microsystem and Information Technology, CAS, Shanghai, 200050 People’s Republic of China; 7 Department of Chemical Engineering and Biotechnology, National Taipei University of Technology (Taipei Tech), 10608 Taipei, Taiwan

**Keywords:** Energy band alignment, X-ray photoelectron spectroscopy, MoS_2_/ZrO_2_, CHF_3_ treatment

## Abstract

The energy band alignment at the multilayer-MoS_2_/ZrO_2_ interface and the effects of CHF_3_ plasma treatment on the band offset were explored using x-ray photoelectron spectroscopy. The valence band offset (VBO) and conduction band offset (CBO) for the MoS_2_ /ZrO_2_ sample is about 1.87 eV and 2.49 eV, respectively. While the VBO was enlarged by about 0.75 eV for the sample with CHF_3_ plasma treatment, which is attributed to the up-shift of Zr 3d core level. The calculation results demonstrated that F atoms have strong interactions with Zr atoms, and the valence band energy shift for the d-orbital of Zr atoms is about 0.76 eV, in consistent with the experimental result. This interesting finding encourages the application of ZrO_2_ as gate materials in MoS_2_-based electronic devices and provides a promising way to adjust the band alignment.

## Introduction

In the past decades, SiO_2_/Si-based materials played the dominant role in the manufacture of electronic devices, such as integrated logic circuits, nonvolatile memory, and so on. However, as the size of the devices scaled down ceaselessly from micrometers to below 10 nm, the traditional semiconductors have been hard to satisfy the requirement of enhanced specific capacitance, low gate leakage current, and high carrier mobility. Therefore, the exploration of new semiconductors as the device channels and the high-κ oxides as insulators becomes agog. Since the discovery of graphene, the successful fabrication of two-dimensional (2D) materials, especially the semiconductors with suitable band gap, has provided a promising way to overcome this drawback.

Among the 2D materials, molybdenum disulfide (MoS_2_) with tunable properties based upon both layer count and the choice of substrate materials has drawn an increasing attention due to not only its good chemical stability and mechanical flexibility but also excellent optical and electrical properties [1, 2]. The energy band gap of the monolayer MoS_2_ is about 1.80 eV while 1.20 eV for bulk. The promising performance of the electronic and optoelectronic devices made from MoS_2_ layers, such as field-effect transistors [3–5], sensors [6], and photodetectors [7], proves it to be potential substitute of Si in conventional electronics and of organic semiconductors in wearable and flexible systems [8–11]. Even though single-layer MoS_2_-based Field-effect transistors (FETs) have exhibited excellent performances with a high current on/off ratio about 10^8^ and a low subthreshold swing ~ 77 mV/decade [3], its extensive applications were hindered by the synthesis of large area high-quality single-layer MoS_2_ and the stability of the devices [12–14]. Multiple-layer MoS_2_ could be more attractive due to the high density of states, which contributes to high drive current in the ballistic limit [15]. In addition, the carrier mobility of multilayer MoS_2_ can be further improved significantly by high-κ oxides owing to the dielectric screening effects [16, 17]. Therefore, it is essential and important to investigate the multilayer MoS_2_/high-κ oxides heterojunctions.

In heterojunction electronic devices, the electron transport properties are precisely controlled by the energy band profiles at the interface between the semiconductor and insulator oxide in the terms of valence band offset (VBO) and conduction band offset (CBO). The VBO and CBO should be as large as possible to operate as a barrier in order to reduce the leakage current formed by the injection of holes and electrons, especially the CBO plays a pivotal role in the selection of suitable high-k oxides for a gate terminal and should be at least larger than 1 eV to avoid current leakage [18–20]. Meanwhile, the interface charges located at semiconductor/oxides impose an important effect on the band engineering and needs to be optimized through passivation technology, such as SiH_4_ passivation, and CHF_3_ treatment. In this paper, we investigated the band alignment of multilayer MoS_2_/ ZrO_2_ systems since the nature of the interface has a direct bearing on the characteristics of the devices, and the effect of CHF_3_ plasma treatment on the band offset at MoS_2_/ZrO_2_ interface was explored.

## Methods and Experiments

In the experiments, the multilayer MoS_2_ films were grown on SiO_2_/Si substrates by chemical vapor deposition (CVD) systems with MoO_3_ and sulfur powder as the Mo sources and S precursors, respectively. During the growth process, Ar gas was used as the carrier gas and the growth temperature was 800 °C for 5 min. Then the MoS_2_/ZrO_2_ samples were prepared by transferring the large area multilayer MoS_2_ film onto the ZrO_2_/Si substrates using the poly methyl methacrylate (PMMA) method. The ZrO_2_ oxide (15 nm) was deposited on Si at 200 °C using atomic layer deposition (BENEQ TFS-200) system with Tetrakis Dimethyl Amido Zirconium (TDMAZr) precursor as the zirconium source and water (H_2_O) as the oxygen source. In order to investigate the effects of CHF_3_ treatment on the band alignment at MoS_2_/ZrO_2_ interfaces, for one sample, the ZrO_2_/Si substrate was treated by CHF_3_ plasma with RF power about 20 W and flow rate about 26 sccm. Meanwhile, the plasma treatment time is about 60 s and the pressure was kept at 1 Pa during the process. Consequently, the resulted F dose is about 2.0 × 10^14^ atoms/cm^2^ estimated by secondary ion mass spectrometry (SIMS) measurements. During the optimization process of the plasma treatment time, the CHF_3_ plasma seriously deteriorated the material quality by introducing fluorine diffused into ZrO_2_ largely when the time was set at 70 s. While when the plasma treatment time was 50 s, smaller than 60 s, SIMS results demonstrated no obvious F peak at the oxide surface. For the other sample, no CHF_3_ plasma treatment was implemented. The Raman characteristics of the samples were taken in a RENISHAW system at room temperature. The X-ray photoelectron spectroscopy (XPS) was measured using a VG ESCALAB 220i-XL system. The photon energy of the monochromatized Al Kα x-ray source is about 1486.6 eV. During the measurements, the pass energy was set at 20 eV in order to obtain the XPS spectra. In addition, C 1 s peak (284.8 eV) was used to correct the core-level binding energy in order to eliminate the sample surface differential charging effect.

## Results and Discussions

**Fig. 1:**
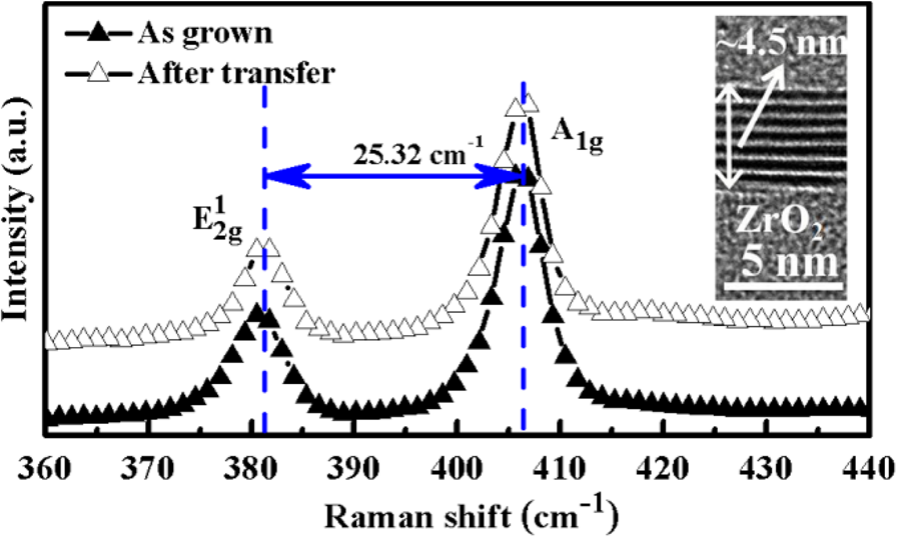
The Raman spectroscopy of the ultrathin MoS_2_ film before and after transfer. The inset is the cross-sectional transmission electron microscopy (TEM) image of the MoS_2_ on ZrO_2_/Si substrate, which shows the layers of MoS_2_

The Raman spectra of the as-grown and after-transferred multilayer MoS_2_ were characterized at room temperature as shown in Fig. 1. Two prominent Raman modes labeled as *A*_1*g*_and ${\mathrm{E}}_{2g}^1$ were observed in the spectrum. Specifically, ${E}_{2g}^1$ mode is resulted from the opposite movement of in-plane S atoms with respect to the central Mo atom in the lower frequency region, whereas *A*_1*g*_ is relative to the out-of-plane vibrations of S atoms in the higher frequency region [21]. It has been observed that the ${\mathrm{E}}_{2g}^1$ and *A*_1*g*_ modes of MoS_2_ undergo a red shift and blue shift, respectively, from monolayer to bulk samples, which is owing to the different interlayer Van der Waals restoring force and the influence of stacking-induced structure changes [21]. Therefore, the frequency difference (Δk) between the *A*_1*g*_ and ${\mathrm{E}}_{2g}^1$ modes is often used to evaluate the layer number or thickness of MoS_2_ film. Herein, Δk of the grown MoS_2_ film is about 25.32 cm^−1^, indicating the film is more than six layers. In addition, the cross-sectional transmission electron microscopy (TEM) result displayed in the inset of Fig. 1 demonstrated the layer number of the grown MoS_2_ was about 8 corresponding to the thickness about 4.5 nm. What is more, the Raman peak position and full width at half maximum (FWHM) of MoS_2_ is almost the same before and after transfer, indicating that the transfer process exerts a small influence on the quality of the material.

**Fig. 2:**
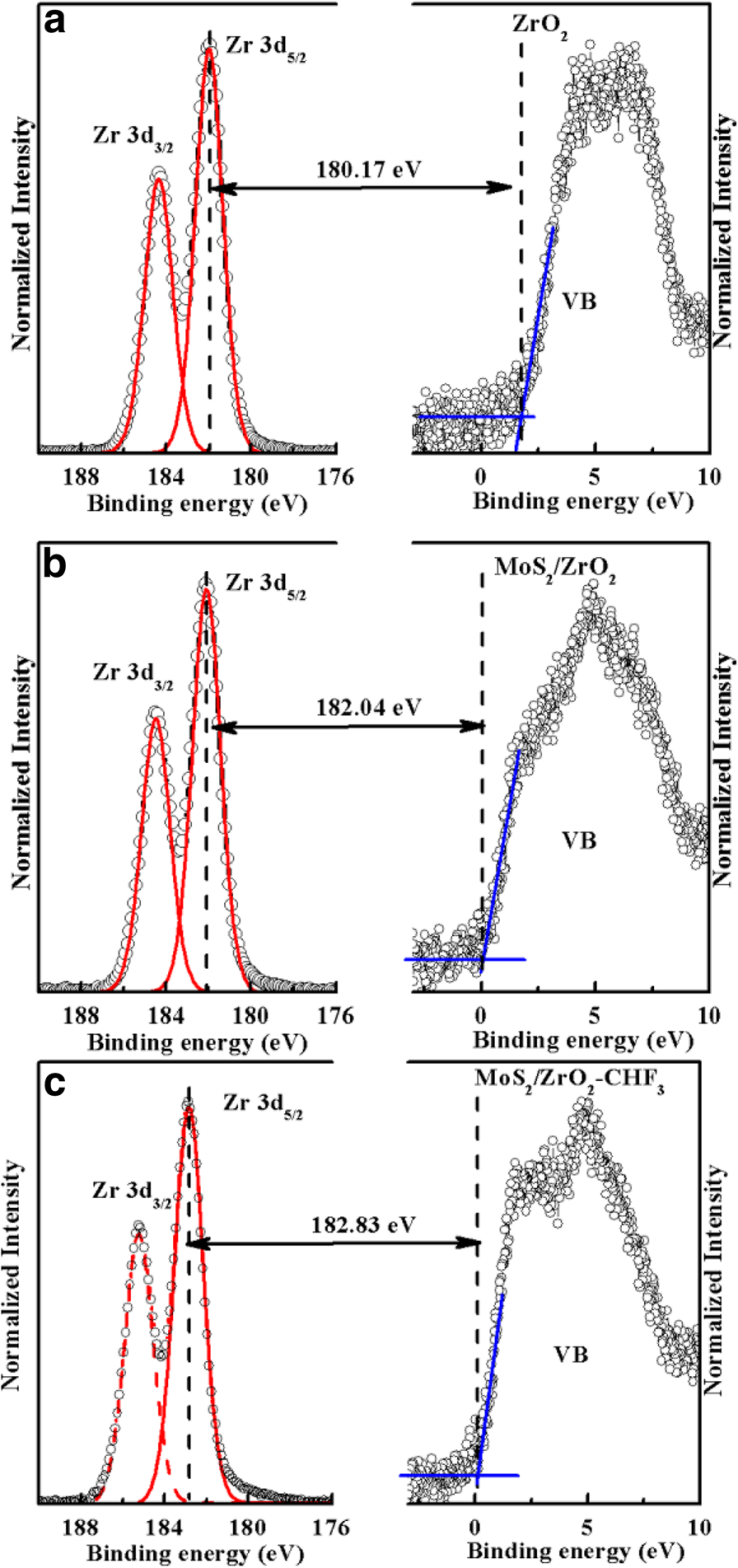
The core-level Zr 3d and valence band spectra for **a** bare ZrO_2_ oxide, **b** multilayer-MoS_2_/ZrO_2_ sample, and **c** CHF_3_ plasma treated multilayer-MoS_2_/ZrO_2_ sample

**Fig. 3:**
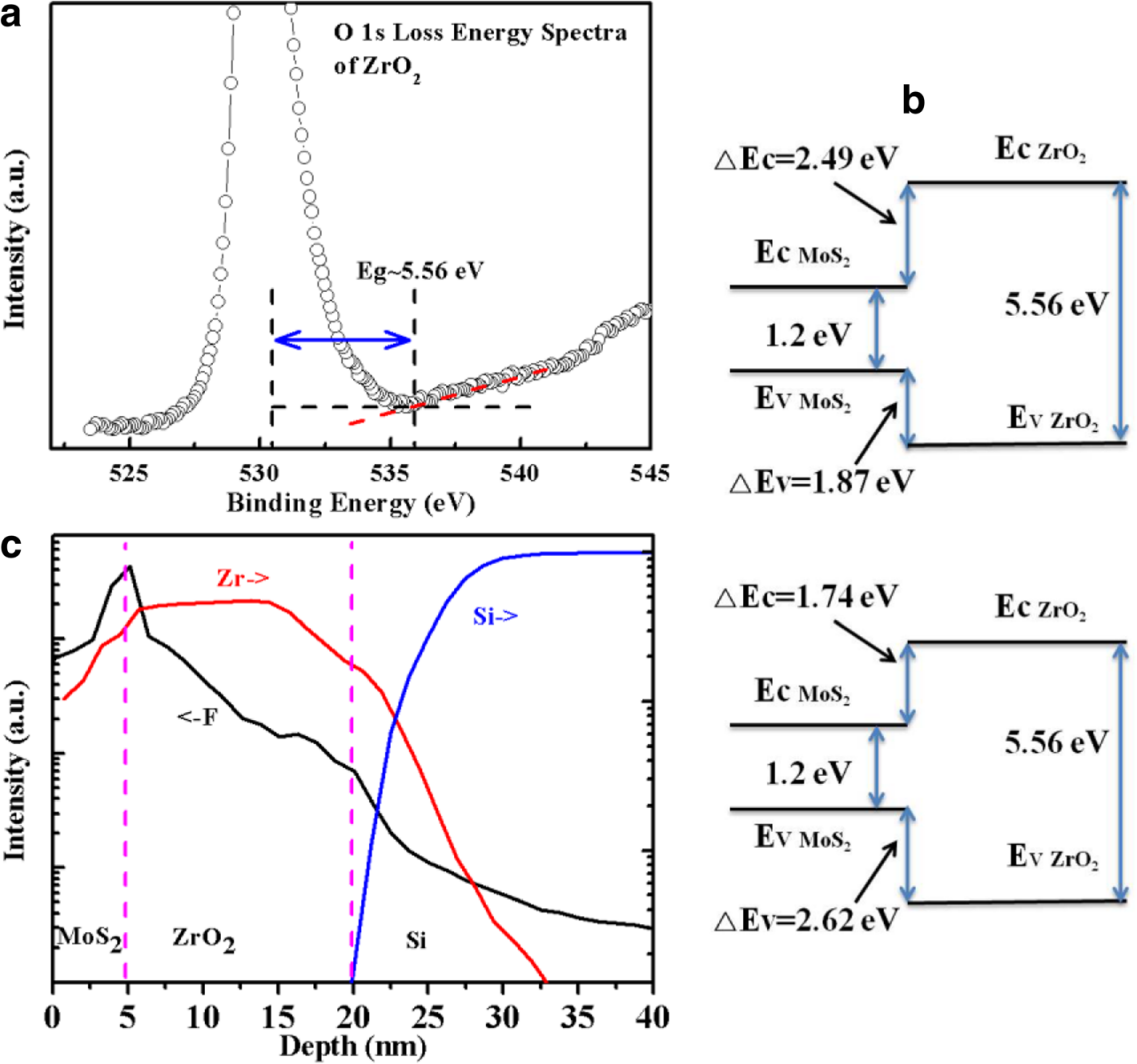
**a** O 1 s photoelectron energy loss spectra for the ZrO_2_ oxide. **b** The schematic structure of the energy band alignment at the MoS_2_/ZrO_2_ interface without (top) and with (bottom) CHF_3_ plasma treatment. **c** The SIMS depth profiles (Si, Zr, and F) for the sample with CHF_3_ plasma treatment

XPS has been profoundly proved to be an efficient way to determine the band offset at the heterojunction interface [22, 23]. In MoS_2_/ZrO_2_ heterojunction, the VBO value was obtained from the change of the valence band spectra of the ZrO_2_ between those of the bare oxide and with MoS_2_ material [24]. Figure 2a, b showed the core level and valance band spectra of bare ZrO_2_ and multilayer-MoS_2_/ZrO_2_, respectively. The intercept between the base line and the slope of the leading edge gives the valence band maximum (VBM) of the sample, where the Fermi level is taken as the reference level. The results demonstrated that the VBM of ZrO_2_ and multilayer-MoS_2_/ZrO_2_ systems are about 1.88 eV and 0.06 eV, respectively. In addition, the Zr 3d core-level spectrum of bare ZrO_2_ exhibits well-separated doublet peaks referred as Zr 3d_5/2_ and 3d_3/2_ with energy values of 182.05 eV and 184.45 eV, respectively, while the corresponding values for the MoS_2_/ZrO_2_ sample are 182.10 eV and 184.50 eV, respectively. The core-level change of Zr 3d_5/2_ or 3d_3/2_ ~ 0.05 eV is in the range of measurement and data processing error. In comparison with bare ZrO_2_ sample, multilayer MoS_2_ exerted little effects on the Zr 3d spectrum as shown in Fig. 2b. Then, the energy difference between the Zr 3d_5/2_ and VBM is 180.17 eV and 182.04 eV for the bare ZrO_2_ sample and MoS_2_/ZrO_2_ sample, respectively. Consequently, the VBO value for the multilayer-MoS_2_/ZrO_2_ interface is about 1.87 ± 0.05 eV, mainly resulted from the VBM difference between the bare ZrO_2_ and MoS_2_/ZrO_2_. Similarly, for the multilayer-MoS_2_/ZrO_2_ sample with CHF_3_ plasma treatment before MoS_2_ transfer, the VBM is about 0.02 eV as shown in Fig. 2c, almost identical to the sample without CHF_3_ treatment. However, the Zr 3d spectrum moves toward higher energy by about 0.75 eV, Zr 3d_5/2_ ~ 182.85 eV, and 3d_3/2_~185.25 eV, indicating that the VBO value was enlarged by about 0.75 ± 0.04 eV after plasma etching. Then, the CBO value *∆E*_*C*_ can be obtained according to the formula

1 $$ \Delta {\mathrm{E}}_{\mathrm{C}}={E}_{G, ZrO2}-{E}_{G, MoS2}-\Delta {E}_V. $$

where *E*_*G*, *ZrO*2_ and *E*_*G*, *MoS*2_ are the band gap of ZrO_2_ and MoS_2_, respectively, and *∆E*_*V*_ corresponds to the VBO value. Normally, the band gap energy of oxide insulator can be obtained from the O 1 s loss energy spectrum [25]. Figure 3a shows the O 1 s loss energy spectrum of ZrO_2_, and the *E*_*G*, *ZrO*2_ is about 5.56 eV calculated from the energy difference by extrapolating the linear edge base line (535.95 eV) fit to the core level energy of Zr-O bonds (530.39 eV). The band gap of MoS_2_ in this work is about 1.2 eV. Therefore, the CBO value for the sample without CHF_3_ treatment is about 2.49 eV and 1.74 eV for the sample with CHF_3_ treatment. Then, the schematic structures of the band engineering for the samples without and with CHF_3_ plasma treatment are illustrated in Fig. 3b. Obviously, the multilayer-MoS_2_/ZrO_2_ system has a type I alignment, which facilitates electrons and holes confined in the MoS_2_. Meanwhile, the large *∆E*_*C*_ and *∆E*_*V*_ for MoS_2_/ZrO_2_ interface implies that ZrO_2_ could be a good gate dielectric for n- or p-channel multilayer MoS_2_-based FETs application in term of gate leakage current suppression. In addition, the sample with plasma treatment has a higher VBO *∆E*_*V*_ (lower CBO *∆E*_*C*_) in comparison with the sample without plasma treatment, which is better in the application of p-channel FETs.

**Fig. 4:**
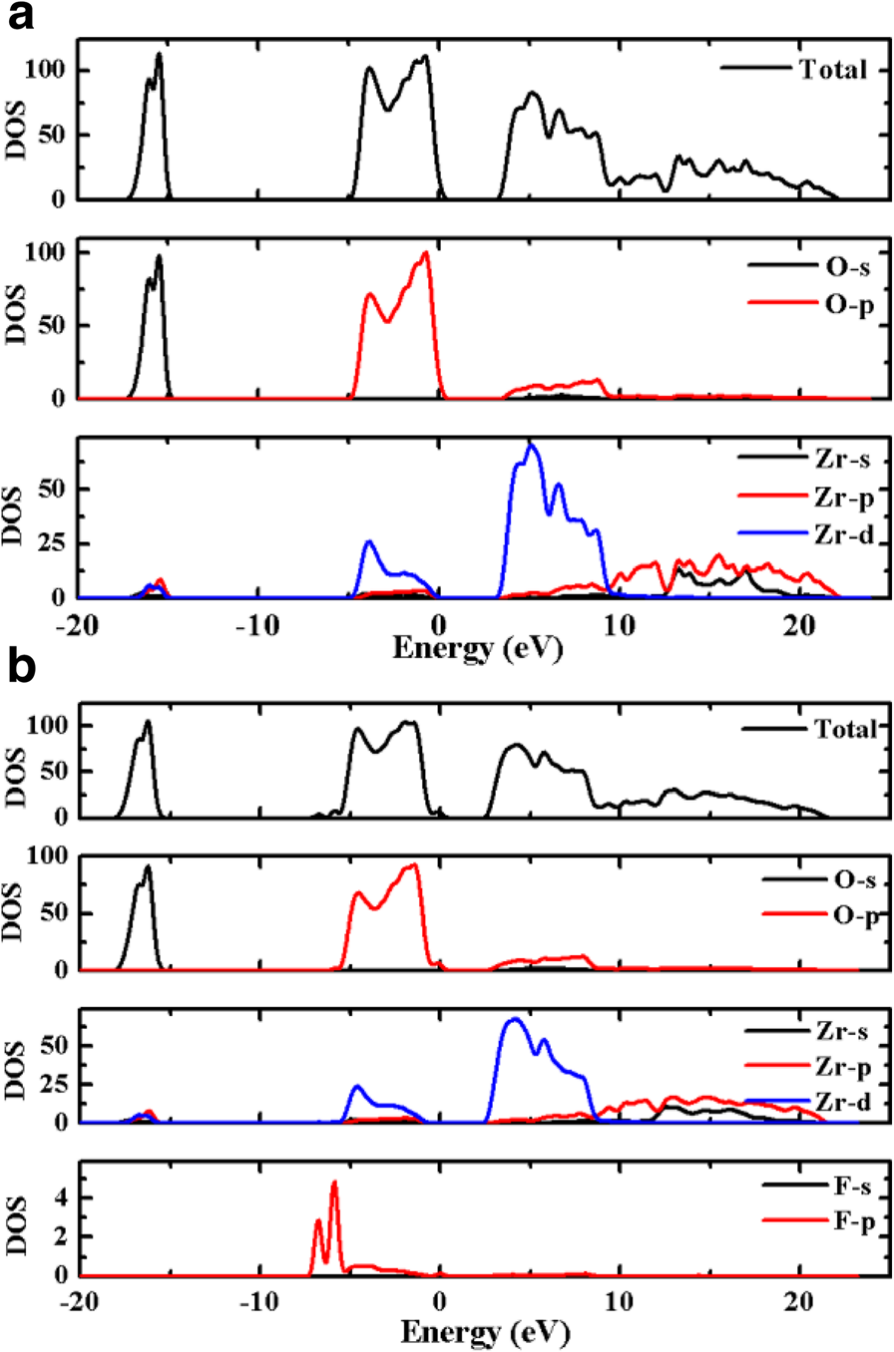
The calculated total density of states (TDOS) and partial density of states (PDOS) for the samples without CHF_3_ treatment (**a**) and with CHF_3_ treatment (**b**)

The change of the band alignment at the multilayer MoS_2_/ZrO_2_ interface is believed to be closely related to the F-rich interfacial layer induced by the CHF_3_ plasma treatment. Figure 3c displayed the SIMS result of the plasma-treated sample for Zr, F, and Si elements, presenting obvious F ions peak at the interface. Meanwhile, some F ions were diffused into the underlying ZrO_2_ layer owing to its small size. At the MoS_2_/ZrO_2_ interface with CHF_3_ plasma treatment, the enlargement of the VBO (reduction of the CBO) is mainly attributed to the up-shift of Zr 3d core-levels shown in Fig. 2c, indicating F ions have a strong interaction with Zr atoms. Then the effects of CHF_3_ treatment on the electronic properties of the ZrO_2_ oxide were investigated using Material Studio combined with the Cambridge Sequential Total Energy Package (CASTEP) based on density functional theory (DFT) [26]. The generalized gradient approximation for the exchange and correlation potential as proposed by Perdew-Burk-Ernzerhof (PBE) [27] was used to treat the ion-electron interactions together with the projector augmented wave potential (PAW) [28]. The plane wave cut-off energy is chosen to be 750 eV, and a Monkhorst–Pack k-mesh of 1 × 1 × 1 is used to sample the Brillouin zone in the structure optimization and total energy calculation [29]. All the atoms were relaxed to their equilibrium positions until the total energy changes during the optimization finally converged to less than 10^−6^ eV*/*atom, the force and stress on each atom was converged to 0.003 eV/nm and 0.05 GPa, respectively, and the displacement was converged to 1 × 10^−4^ nm. Figure 4a, b shows the total and partial density of states (DOS) for both MoS_2_/ZrO_2_ samples, where zero eV corresponds to the Fermi level. Obviously, F ions have a strong interaction with Zr atoms, making part of the d-orbital from Zr atoms which is projected to valence band moves downward about 0.76 eV from − 0.06 to − 0.82 eV below the Fermi level, which is in consistent with the enlargement of the valance band offset *∆E*_*V*_ ~ 0.75 eV. F atoms tend to attract electrons owing to the large electronegativity (4.0) and become partially negatively charged and then further form dipoles with Zr atoms, eventually contribute to the change of the band offset. Therefore, the band change at the MoS_2_/ZrO_2_ interface introduced by the CHF_3_ plasma treatment provides a promising way to adjust the band alignment at the heterojunctions, which facilitates the design of the related devices.

## Conclusions

In this paper, we explored the energy band engineering at the multilayer MoS_2_/ZrO_2_ interface and investigated the effects of CHF_3_ treatment using x-ray photoelectron spectroscopy. The results demonstrated that a type I alignment was formed at the MoS_2_/ZrO_2_ heterojunction interface with CBO and VBO about 2.49 eV and 1.87 eV, respectively. While the CHF_3_ plasma treatment increases the VBO by about 0.75 ± 0.04 eV mainly due to the up-shift of Zr 3d core-level energy, which is consistent with the calculation results. This work proves the great potential applications of high-κ ZrO_2_ oxide in multilayer MoS_2_-based devices and provides a possible way to modify the interface energy band alignment.

## Abbreviations

2D: Two-dimensional
CASTEP: Cambridge Sequential Total Energy Package
CBO: Conduction band offset
CVD: Chemical vapor deposition
DFT: Density functional theory
DOS: Density of states
FETs: Field-effect transistors
FWHM: Full width at half maximum
MoS_2_: Molybdenum disulphide
PAW: Projector augmented wave
PBE: Perdew-Burk-Ernzerhof
PMMA: Poly methyl methacrylate
SIMS: Secondary ion mass spectrometry
TDMAZr: Tetrakis Dimethyl Amido Zirconium
TEM: Transmission electron microscopy
TMDs: Transition metal dichalcogenides
VBO: Valence band offset
XPS: X-ray photoelectron spectroscopy
ZrO_2_: Zirconium dioxide
